# A collection of metagenome-assembled genomes from platinum mine tailings in South Africa

**DOI:** 10.1128/mra.00569-25

**Published:** 2025-09-23

**Authors:** Monono Mobanga, Ayomide Emmanuel Fadiji, Kalonji Abondance Tshisekedi, Tonderayi Dave Kawadza, Olubukola Oluranti Babalola

**Affiliations:** 1 Food Security and Safety Focus Area, Faculty of Natural and Agricultural Sciences, North-West University 274472 , Mmabatho, South Africa; 2 Hawkesbury Institute for the Environment, Western Sydney University 89380, Richmond, New South Wales, Australia; 3 Sydney Brenner Institute for Molecular Bioscience, Faculty of Health Sciences, University of the Witwatersrand 587870, Johannesburg, South Africa; Rochester Institute of Technology, Rochester, New York, USA

**Keywords:** platinum mine tailings, heavy metals, bioremediation, metagenomics

## Abstract

This study presents 84 metagenome-assembled genomes from platinum mine tailings and non-mined soil. The analysis reveals diverse Proteobacteria. This work offers insights into pollutant degradation, resource recovery, and potential strategies for ecological restoration in mining-impacted environments.

## ANNOUNCEMENT

The mining industry in South Africa draws upon vast platinum, gold, coal, and diamond reserves (1). However, mining introduces toxic metals, suppresses soil regeneration, and challenges microbial survival (2–4). Many soil microbes remain uncultured, so metagenomics is crucial for revealing their diversity and functions, informing strategies for remediation, metal recovery, and sustainable mining (5–9).

We collected tailings dam samples from two platinum mines on the western limb of the Bushveld Igneous Complex, one 30 km northwest of Rustenburg in the North West Province (B) and the other 30 km south of Thabazimbi in Limpopo Province (N). Samples of the tailings were collected from the surface (10–15 cm deep) of each tailings dam using aseptic techniques, and soil samples (BC, NC) were also collected from non-mining areas. Genomic DNA was extracted from 0.25 g of each sample using the DNeasy PowerSoil kit (MO BIO Laboratories, USA). Sample integrity was verified with the Agilent 5400 fragment analyzer. Genomic DNA was sheared, end-repaired, A-tailed, adapter-ligated, PCR-amplified, size-selected, and purified. Libraries were prepared using the NEBNext Ultra II library preparation kit as per manufacturer’s instructions and were quantified (Qubit, qPCR, bioanalyzer), pooled, and sequenced on an Illumina MiSeq PE 250 bp platform by Novogene (Singapore).

**Fig 1:**
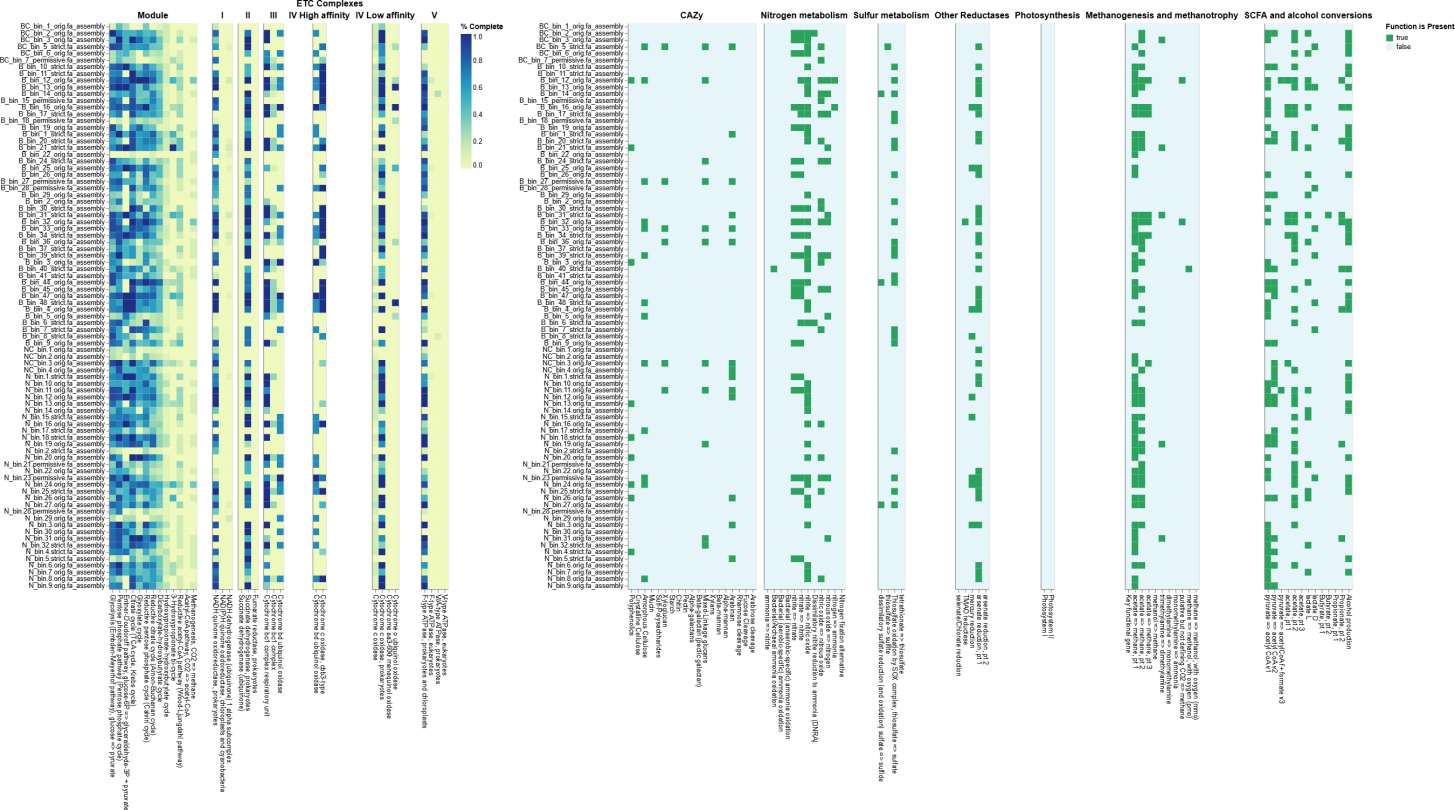
DRAM annotations of the MAGs recovered from platinum mine tailings in South Africa.

Metagenome sequencing yielded a total of 181,695,000 paired-end reads across all samples (range: 18,914,000–27,873,000 reads per sample). Low-quality reads and adapters were removed with Trimmomatic v0.39 (5). Cleaned reads were taxonomically classified using Kraken2 v2.1.1 and Bracken v2.6.2 (10). Identified taxa were dominated by Bacteria, with minor proportions of Archaea, Eukarya, and Viruses. Quality-filtered reads were assembled via metaSPAdes v3.15.3 (11). Contigs ≥1,000 bp were binned using MaxBin v2.0 (12), metaBAT2 v2.8 (13), and CONCOCT v1.0.07 (14), then refined with MetaWRAP v1.3.2 (15). CheckM v1.2.218 was used to evaluate the completeness of the metagenome-assembled genomes (MAGs) and all demonstrated ≥50% completeness and ≤10% contamination (16), 42 of the 84 MAGs had high completeness levels (≤80%). The retained bins underwent phylogenomic analysis and taxonomic assignment via GTDB-tk v1.749 (17) and were functionally annotated with DRAM v0.1.2 (18) (Fig. 1). Default parameters were used for all software, unless otherwise stated. The recovered MAGs spanned 15 bacterial classes and 48 genera, whereas the read-base analysis spanned 13 bacterial classes and 67 genera (Table 1). From both analyses, *Pseudomonas* had higher abundance levels reflecting its pollutant-degrading capacity (19).

**TABLE 1:** Features and accession numbers of the MAGs from platinum tailings and soil samples

MAG_Name	GTDB-Tk classification	Size (Mbp)	No. of contigs	N50 (Mbp)	GC content (%)	No. of CDS^a^	Comp/ Cont (%)^b^	NCBI SRA Accession no.^c^	NCBI Genbank accession no.
NC_bin.4_Soil	Bacteria; Actinobacteriota; Actinomycetia; Propionibacteriales; Propionibacteriaceae;	2.03	674	3,291.0	72.4	2,318	59.74/0.0	SRR23730087 SRR23730088	JBKHYK000000000
NC_bin.3_Soil	Bacteria; Acidobacteriota; Blastocatellia; UBA7656; UBA7656; JADGNW01	3.59	134	62,162.0	72.7	5,500	93.88/5.55	SRR23730087 SRR23730088	JBKHYJ000000000
NC_bin.2_Soil	Bacteria; Acidobacteriota; Thermoanaerobaculia; UBA5704; UBA5704; UBA5704	3.44	2,086	3,489.0	68.8	5,333	67.67/2.84	SRR23730087 SRR23730088	JBKHYI000000000
NC_bin.1_Soil	Bacteria; Acidobacteriota; Thermoanaerobaculia; Gp7-AA8; Gp7-AA8; QHVT01	1.79	1,155	2,557.0	68.1	3,181	56.52/1.79	SRR23730087 SRR23730088	JBKHYH000000000
N_bin.9_Tailings	Bacteria; Actinobacteriota; UBA4738; AC-67; AC-67;	2.04	135	27,483.0	64.0	2,062	97.09/0.85	SRR23730085 SRR23730086	JBKHYG000000000
N_bin.8_Tailings	Bacteria; Proteobacteria; Gammaproteobacteria; Nevskiales; Nevskiaceae; Fontimonas	3.5	976	3,983	68.8	3,840	72.57/2.58	SRR23730085 SRR23730086	JBKHYF000000000
N_bin.7_Tailings	Bacteria; Actinobacteriota; Actinomycetia; Mycobacteriales; SCTD01;	2.6	187	17,651.0	74.0	2,586	65.51/0.0	SRR23730085 SRR23730086	JBKHYE000000000
N_bin.6_Tailings	Bacteria; Actinobacteriota; Acidimicrobiia; Acidimicrobiales; JACDCH01;	3.24	75	109,669.0	72.8	3,160	94.01/4.7	SRR23730085 SRR23730086	JBKHYD000000000
N_bin.5_Tailings	Bacteria; Actinobacteriota; Actinomycetia; Propionibacteriales; Nocardioidaceae; Nocardioides_B	2.0	210	30,316.0	71.6	2,072	54.31/0.0	SRR23730085 SRR23730086	JBKHYC000000000
N_bin.4_Tailings	Bacteria; Actinobacteriota; UBA4738; CADDZG01; WHSQ01;	1.93	427	5,789.0	63.9	2,253	78.04/2.73	SRR23730085 SRR23730086	JBKHYB000000000
N_bin.32_Tailings	Bacteria; Actinobacteriota; Actinomycetia; Actinomycetales; Microbacteriaceae; Yonghaparkia	1.91	210	23,569.0	71.8	1,996	80.89/2.81	SRR23730085 SRR23730086	JBKHYA000000000
N_bin.31_Tailings	Bacteria; Actinobacteriota; Acidimicrobiia; UBA5794; ZC4RG35;	2.75	313	14,481.0	65.8	2,977	92.54/1.28	SRR23730085 SRR23730086	JBKHXZ000000000
N_bin.30_Tailings	Bacteria; Actinobacteriota; Actinomycetia; Actinomycetales; Microbacteriaceae; Agrococcus Agrococcus sediminis	1.36	178	8,318.0	73.4	1,438	59.28/0.25	SRR23730085 SRR23730086	JBKHXY000000000
N_bin.3_Tailings	Bacteria; Actinobacteriota; Acidimicrobiia; Acidimicrobiales; AC-14;	2.87	95	62,162.0	72.0	3,591	71.44/1.21	SRR23730085 SRR23730086	JBKHXX000000000
N_bin.29_Tailings	Bacteria; Proteobacteria; Alphaproteobacteria; Caulobacterales; Caulobacteraceae; Brevundimonas Brevundimonas sp001794825	2.63	485	2,670.0	70.2	1,696	57.7/6.41	SRR23730085 SRR23730086	JBKHXW000000000
N_bin.28_Tailings	Bacteria; Acidobacteriota; Vicinamibacteria; Vicinamibacterales; UBA2999;	2.19	918	2,692.0	71.7	2,564	53.44/1.72	SRR23730085 SRR23730086	JBKHXV000000000
N_bin.27_Tailings	Bacteria; Proteobacteria; Gammaproteobacteria; Acidiferrobacterales; Sulfurifustaceae;	2.65	251	15,632.0	63.6	2,751	95.38/1.43	SRR23730085 SRR23730086	JBKHXU000000000
N_bin.26_Tailings	Bacteria; Actinobacteriota; Acidimicrobiia; Acidimicrobiales; JACDCH01;	2.8	229	14,640.0	73.8	2,844	78.63/1.7	SRR23730085 SRR23730086	JBKHXT000000000
N_bin.25_Tailings	Bacteria; Proteobacteria; Gammaproteobacteria; Burkholderiales; Burkholderiaceae; Hydrogenophaga	2.67	367	11,962.0	65.6	2,877	79.67/1.19	SRR23730085 SRR23730086	JBKHXS000000000
N_bin.24_Tailings	Bacteria; Actinobacteriota; Thermoleophilia; Solirubrobacterales; Solirubrobacteraceae; CADCVQ01	4.21	122	50,696.0	72.6	4,194	98.29/4.72	SRR23730085 SRR23730086	JBKHXR000000000
N_bin.23_Tailings	Bacteria; Proteobacteria; Gammaproteobacteria; Pseudomonadales; Pseudomonadaceae; Pseudomonas_A Pseudomonas_A sp003205815	4.1	932	7,399.0	64.5	4,565	65.51/6.89	SRR23730085 SRR23730086	JBKHXQ000000000
N_bin.22_Tailings	Bacteria; Actinobacteriota; Acidimicrobiia; Acidimicrobiales; CADCTF01;	2.25	724	3,383.0	68.7	2,692	62.61/2.56	SRR23730085 SRR23730086	JBKHXP000000000
N_bin.21_Tailings	Bacteria; Actinobacteriota; Actinomycetia; Actinomycetales; Micrococcaceae; Pseudarthrobacter Pseudarthrobacter phenanthrenivorans	3.34	1392	2,916.0	66.4	4,010	54.88/0.0	SRR23730085 SRR23730086	JBKHXO000000000
N_bin.20_Tailings	Bacteria; Proteobacteria; Gammaproteobacteria; Nevskiales; Nevskiaceae;	2.1	140	25,891.0	68.1	2,068	84.48/0.0	SRR23730085 SRR23730086	JBKHXN000000000
N_bin.2_Tailings	Bacteria; Proteobacteria; Gammaproteobacteria; SURF-13; SURF-13; SURF-13 SURF-13 sp003599485	1.12	449	2,841.0	62.6	1,418	69.93/2.22	SRR23730085 SRR23730086	JBKHXM000000000
N_bin.19_Tailings	Bacteria; Chloroflexota; Limnocylindria; Limnocylindrales; CSP1-4; SPCO01	2.13	121	33,539.0	72.9	2,785	87.64/0.0	SRR23730085 SRR23730086	JBKHXL000000000
N_bin.18_Tailings	Bacteria; Actinobacteriota; UBA4738; CADDZG01; WHSQ01; MB1-2	2.5	99	62,812.0	65.4	2,614	95.72/0.15	SRR23730085 SRR23730086	JBKHXK000000000
N_bin.17_Tailings	Bacteria; Gemmatimonadota; Gemmatimonadetes; Gemmatimonadales; Gemmatimonadaceae; SCN-70-22	3.13	1064	3,496.0	69.7	3,392	67.48/0.83	SRR23730085 SRR23730086	JBKHXJ000000000
N_bin.16_Tailings	Bacteria; Proteobacteria; Gammaproteobacteria; Xanthomonadales; Xanthomonadaceae; Vulcaniibacterium_B	2.0	243	10,585.0	72.8	1,898	83.4/1.51	SRR23730085 SRR23730086	JBKHXI000000000
N_bin.15_Tailings	Bacteria; Actinobacteriota; Actinomycetia; Actinomycetales; Micrococcaceae; Arthrobacter_D Arthrobacter_D subterraneus	2.93	342	17,640.0	65.5	2,956	90.51/3.26	SRR23730085 SRR23730086	JBKHXH000000000
N_bin.14_Tailings	Bacteria; Actinobacteriota; Actinomycetia; Mycobacteriales; Geodermatophilaceae; Blastococcus	2.79	1541	9,834.0	67.1	4,226	66.72/1.72	SRR23730085 SRR23730086	JBKHXG000000000
N_bin.13_Tailings	Bacteria; Actinobacteriota; Actinomycetia; Nitriliruptorales; ;	3.11	37	113,821.0	74.5	2,863	84.61/0.85	SRR23730085 SRR23730086	JBKHXF000000000
N_bin.12_Tailings	Bacteria; Actinobacteriota; Actinomycetia; Actinomycetales; Quadrisphaeraceae;	3.31	187	22,874.0	74.1	3,229	88.26/1.42	SRR23730085 SRR23730086	JBKHXE000000000
N_bin.11_Tailings	Bacteria; Actinobacteriota; Actinomycetia; Actinomycetales; Demequinaceae; Demequina	2.24	241	12,269.0	70.6	2,245	88.39/1.73	SRR23730085 SRR23730086	JBKHXD000000000
N_bin.10_Tailings	Bacteria; Actinobacteriota; Acidimicrobiia; Acidimicrobiales; GCA-2861595;	2.83	573	6,312.0	76.0	2,999	75.06/4.34	SRR23730085 SRR23730086	JBKHXC000000000
N_bin.1_Tailings	Bacteria; Proteobacteria; Alphaproteobacteria; Sphingomonadales; Sphingomonadaceae; Sphingomicrobium	3.68	195	75,733.0	71.8	2,251	93.58/2.56	SRR23730085 SRR23730086	JBKHXB000000000
BC_bin_7_Soil	Bacteria; Proteobacteria; Gammaproteobacteria; Burkholderiales; Burkholderiaceae; SCN-69-89	2.32	1405	1,755.0	67.7	3,081	57.37/3.98	SRR23730084 SRR23730089	JBKHXA000000000
BC_bin_6_Soil	Bacteria; Actinobacteriota; Actinomycetia; Actinomycetales; Micrococcaceae; Pseudarthrobacter	2.5	1242	2,168.0	66.6	3,025	71.03/3.21	SRR23730084 SRR23730089	JBKHWZ000000000
BC_bin_5_Soil	Bacteria; Proteobacteria; Gammaproteobacteria; Burkholderiales; Burkholderiaceae; Massilia	4.81	885	8295.0	65.9	4,743	83.32/2.57	SRR23730084 SRR23730089	JBKHWY000000000
BC_bin_3_Soil	Bacteria; Actinobacteriota; Actinomycetia; Actinomycetales; Quadrisphaeraceae;	7.35	309	95,072.0	60.1	2,857	98.29/5.98	SRR23730084 SRR23730089	JBKHWX000000000
BC_bin_2_Soil	Bacteria; Actinobacteriota; Actinomycetia; Propionibacteriales; Propionibacteriaceae; Brevilactibacter	4.73	197	16039.0	69.5	2,303	52.26/6.35	SRR23730084 SRR23730089	JBKHWW000000000
BC_bin_1_Soil	Bacteria; Actinobacteriota; Thermoleophilia; Solirubrobacterales; 70-9;	2.72	747	2,375.0	65.6	2,270	50.64/1.7	SRR23730084 SRR23730089	JBKHWV000000000
B_bin_9_Tailings	Bacteria; Proteobacteria; Gammaproteobacteria; Burkholderiales; Burkholderiaceae; SCN-69-89 SCN-69-89 sp001724315	2.03	287	8,573.0	70.6	2,082	70.06/2.16	SRR23730090 SRR23730091	JBKHWU000000000
B_bin_8_Tailings	Bacteria; Proteobacteria; Gammaproteobacteria; Burkholderiales; Thiobacillaceae; Thiobacillus	1.81	318	11,559.0	67.1	2,011	65.39/7.63	SRR23730090 SRR23730091	JBKHWT000000000
B_bin_7_Tailings	Bacteria; Bdellovibrionota; Bacteriovoracia; Bacteriovoracales; Bacteriovoracaceae; UBA4096	3.1	174	44,707.0	42.7	3,256	83.18/0.89	SRR23730090 SRR23730091	JBKHWS000000000
B_bin_6_Tailings	Bacteria; Proteobacteria; Gammaproteobacteria; Burkholderiales; Thiobacillaceae; Thiobacillus	1.75	188	33,998.0	65.7	1,827	64.26/1.79	SRR23730090 SRR23730091	JBKHWR000000000
B_bin_5_Tailings	Bacteria; Proteobacteria; Gammaproteobacteria; Burkholderiales; Burkholderiaceae; Acidovorax Acidovorax sp001411535	4.15	2196	2,000.0	64.3	5,283	72.71/3.87	SRR23730090 SRR23730091	JBKHWQ000000000
B_bin_48_Tailings	Bacteria; Proteobacteria; Gammaproteobacteria; Pseudomonadales; Pseudomonadaceae; Pseudomonas_E Pseudomonas_E sp014268815	5.49	468	21,4476.0	61.9	5,382	84.78/1.43	SRR23730090 SRR23730091	JBKHWP000000000
B_bin_47_Tailings	Bacteria; Proteobacteria; Alphaproteobacteria; Sphingomonadales; Sphingomonadaceae; Novosphingobium	2.21	61	61,685.0	62.9	2,170	97.8/0.33	SRR23730090 SRR23730091	JBKHWO000000000
B_bin_45_Tailings	Bacteria; Proteobacteria; Gammaproteobacteria; Pseudomonadales; JADFDG01; JADFDG01	2.06	210	11,887.0	65.6	2,008	66.37/0.0	SRR23730090 SRR23730091	JBKHWN000000000
B_bin_44_Tailings	Bacteria; Proteobacteria; Gammaproteobacteria; Burkholderiales; Burkholderiaceae; Calidifontimicrobium	3.7	323	16,194.0	72.8	3,649	94.81/2.57	SRR23730090 SRR23730091	JBKHWM000000000
B_bin_41_Tailings	Bacteria; Proteobacteria; Gammaproteobacteria; Burkholderiales; Thiobacillaceae; Thiobacillus	1.54	224	10,494.0	67.9	1,733	64.38/1.63	SRR23730090 SRR23730091	JBKHWL000000000
B_bin_40_Tailings	Bacteria; Proteobacteria; Gammaproteobacteria; Pseudomonadales; Porticoccaceae; JAGPUQ01	1.89	199	17,365.0	63.6	1,901	78.45/0.92	SRR23730090 SRR23730091	JBKHWK000000000
B_bin_4_Tailings	Bacteria; Proteobacteria; Gammaproteobacteria; Pseudomonadales; Pseudomonadaceae; Pseudomonas_E Pseudomonas_E peli	4.61	158	56,147.0	59.9	4,449	96.35/0.36	SRR23730090 SRR23730091	JBKHWJ000000000
B_bin_39_Tailings	Bacteria; Proteobacteria; Gammaproteobacteria; Burkholderiales; Thiobacillaceae; Thiobacillus	2.3	173	36,904.0	63.1	2,344	65.86/3.44	SRR23730090 SRR23730091	JBKHWI000000000
B_bin_37_Tailings	Bacteria; Proteobacteria; Gammaproteobacteria; Burkholderiales; Thiobacillaceae; Thiobacillus	1.61	134	32,181.0	64.6	1,693	56.51/3.81	SRR23730090 SRR23730091	JBKHWH000000000
B_bin_36_Tailings	Bacteria; Proteobacteria; Alphaproteobacteria; Sphingomonadales; Sphingomonadaceae; Sphingobium Sphingobium sp001713415	3.91	1533	2,980.0	63.0	4,754	82.57/3.68	SRR23730090 SRR23730091	JBKHWG000000000
B_bin_34_Tailings	Bacteria; Proteobacteria; Alphaproteobacteria; Caulobacterales; Caulobacteraceae; Phenylobacterium Phenylobacterium sp002441055	2.99	119	52,743.0	67.4	3,026	96.32/1.29	SRR23730090 SRR23730091	JBKHWF000000000
B_bin_33_Tailings	Bacteria; Bacteroidota; Bacteroidia; Flavobacteriales; Flavobacteriaceae; Flavobacterium	4.3	24	415,372.0	43.2	3,729	99.29/0.35	SRR23730090 SRR23730091	JBKHWE000000000
B_bin_32_Tailings	Bacteria; Proteobacteria; Gammaproteobacteria; Burkholderiales; Rhodocyclaceae; Thauera	2.89	176	27,186.0	68.8	2,774	86.99/5.25	SRR23730090 SRR23730091	JBKHWD000000000
B_bin_31_Tailings	Bacteria; Proteobacteria; Alphaproteobacteria; Rhodobacterales; Rhodobacteraceae; Tabrizicola	3.5	201	34,443.0	67.2	3,653	93.86/1.0	SRR23730090 SRR23730091	JBKHWC000000000
B_bin_30_Tailings	Bacteria; Proteobacteria; Gammaproteobacteria; Pseudomonadales; Moraxellaceae; UBA2031	2.17	185	21,864.0	62.2	2,157	64.13/0.0	SRR23730090 SRR23730091	JBKHWB000000000
B_bin_3_Tailings	Bacteria; Proteobacteria; Gammaproteobacteria; Pseudomonadales; JADFDG01; JADFDG01	2.22	182	17,739.0	67.6	2,157	62.06/0.0	SRR23730090 SRR23730091	JBKHWA000000000
B_bin_29_Tailings	Bacteria; Actinobacteriota; Acidimicrobiia; Acidimicrobiales; Ilumatobacteraceae;	1.34	1007	2,869.0	71.2	3,175	52.99/1.99	SRR23730090 SRR23730091	JBKHVZ000000000
B_bin_28_Tailings	Bacteria; Bdellovibrionota; Bacteriovoracia; Bacteriovoracales; Bacteriovoracaceae; UBA6144	2.64	421	10,321.0	56.1	2,880	81.74/1.78	SRR23730090 SRR23730091	JBKHVY000000000
B_bin_27_Tailings	Bacteria; Bacteroidota; Bacteroidia; Sphingobacteriales; Sphingobacteriaceae; Pedobacter	3.65	940	5,050.0	39.2	3,875	87.22/1.15	SRR23730090 SRR23730091	JBNQYF000000000
B_bin_26_Tailings	Bacteria; Proteobacteria; Gammaproteobacteria; Burkholderiales; Burkholderiaceae; Hydrogenophaga	2.17	260	9,728.0	65.0	2,246	67.46/3.97	SRR23730090 SRR23730091	JBKHVX000000000
B_bin_25_Tailings	Bacteria; Proteobacteria; Alphaproteobacteria; Sphingomonadales; Sphingomonadaceae; Rhizorhabdus Rhizorhabdus sp018006725	3.02	737	5,123.0	65.8	3,274	81.71/4.07	SRR23730090 SRR23730091	JBKHVW000000000
B_bin_24_Tailings	Bacteria; Bacteroidota; Bacteroidia; Flavobacteriales; UBA10066	2.03	180	21,039.0	35.7	2,056	80.65/1.43	SRR23730090 SRR23730091	JBKHVV000000000
B_bin_22_Tailings	Bacteria; Proteobacteria; Alphaproteobacteria; UBA11222; UBA11222; UBA11222	1.43	773	1,970.0	57.6	1,871	57.66/1.08	SRR23730090 SRR23730091	JBKHVU000000000
B_bin_21_Tailings	Bacteria; Proteobacteria; Alphaproteobacteria; Rhodobacterales; Rhodobacteraceae; EhC02	3.94	253	37,958.0	64.1	4,015	97.8/1.61	SRR23730090 SRR23730091	JBKHVT000000000
B_bin_20_Tailings	Bacteria; Proteobacteria; Gammaproteobacteria; Burkholderiales; Burkholderiaceae; Quisquiliibacterium	3.24	388	16,534.0	70.9	3,272	87.79/2.11	SRR23730090 SRR23730091	JBKHVS000000000
B_bin_2_Tailings	Bacteria; Cyanobacteria; Sericytochromatia	2.39	1083	16,039.000000000002	70.2	3,752	80.22/1.33	SRR23730090 SRR23730091	JBKHVR000000000
B_bin_19_Tailings	Bacteria; Actinobacteriota; Thermoleophilia; Miltoncostaeales; Miltoncostaeaceae;	2.89	427	6,178.0	72.7	2,402	91.82/1.08	SRR23730090 SRR23730091	JBKHVQ000000000
B_bin_18_Tailings	Bacteria; Proteobacteria; Gammaproteobacteria; Burkholderiales; Burkholderiaceae; Limnobacter Limnobacter sp001601825	1.85	1016	1,927.0	53.2	2,430	61.98/2.96	SRR23730090 SRR23730091	JBKHVP000000000
B_bin_17_Tailings	Bacteria; Proteobacteria; Gammaproteobacteria; Burkholderiales; Burkholderiaceae; Giesbergeria Giesbergeria sp001770955	2.62	393	11,437.0	66.0	2,676	65.51/0.0	SRR23730090 SRR23730091	JBKHVO000000000
B_bin_16_Tailings	Bacteria; Proteobacteria; Gammaproteobacteria; Burkholderiales; Burkholderiaceae; Pseudacidovorax Pseudacidovorax sp903864295	6.04	837	9,544.0	69.3	6,139	93.79/2.7	SRR23730090 SRR23730091	JBKHVN000000000
B_bin_15_Tailings	Bacteria; Bdellovibrionota; Bdellovibrionia; Bdellovibrionales; Bdellovibrionaceae	2.57	141	33,321.0	41.5	2,540	95.55/0.9	SRR23730090 SRR23730091	JBKHVM000000000
B_bin_14_Tailings	Bacteria; Proteobacteria; Gammaproteobacteria; JAHJQQ01; JAHJQQ01	3.36	427	2,465.0	73.6	2,982	64.57/8.15	SRR23730090 SRR23730091	JBKHVL000000000
B_bin_13_Tailings	Bacteria; Proteobacteria; Gammaproteobacteria; Pseudomonadales; Pseudomonadaceae; Pseudomonas_E Pseudomonas_E sp002874965	6.98	218	5,6275.0	61.0	6,394	97.03/1.61	SRR23730090 SRR23730091	JBKHVK000000000
B_bin_12_Tailings	Bacteria; Proteobacteria; Alphaproteobacteria; Azospirillales; Azospirillaceae; Azospirillum	7.19	501	19,224.0	67.6	6,914	93.49/4.09	SRR23730090 SRR23730091	JBKHVJ000000000
B_bin_11_Tailings	Bacteria; Bdellovibrionota; Bacteriovoracia; Bacteriovoracales; Bacteriovoracaceae; UBA6144	3.01	131	41,998.0	44.6	3,142	82.06/0.94	SRR23730090 SRR23730091	JBKHVI000000000
B_bin_10_Tailings	Bacteria; Proteobacteria; Gammaproteobacteria; Immundisolibacterales; Immundisolibacteraceae; Immundisolibacter	2.64	436	11,746.0	68.2	2,800	72.75/0.0	SRR23730090 SRR23730091	JBKHVH000000000
B_bin_1_Tailings	Bacteria; Actinobacteriota; Acidimicrobiia; Acidimicrobiales; JACDCH01	2.06	219	76,480.0	65.8	3,795	90.93/1.67	SRR23730090 SRR23730091	JBKHVG000000000

^a^ CDS, coding sequences.

^b^ SRA accession number for the raw metagenomic reads for each MAG.

^c^ Compl., completeness value; Cont., contamination value.

## Data Availability

All sequence data are deposited at the NCBI under BioProject PRJNA942043. Raw sequencing data for each sample are available under accession numbers SRX19590709 to SRX19590712, and the MAGs are available under GenBank accession numbers JBNQYF000000000; JBKHVG000000000 to JBKHYK000000000).

## References

[1] Rauch S, Fatoki OS. 2013. Anthropogenic platinum enrichment in the vicinity of mines in the bushveld igneous complex, South Africa. Water Air Soil Pollut 224:1395. doi:10.1007/s11270-012-1395-y

[2] Artiola J, Pepper IL, Brusseau ML. 2004. Environmental monitoring and characterization. Elsevier Science.

[3] Dopson M, Johnson DB. 2012. Biodiversity, metabolism and applications of acidophilic sulfur-metabolizing microorganisms. Environ Microbiol 14:2620–2631. doi:10.1111/j.1462-2920.2012.02749.x 22510111

[4] Chung AP, Coimbra C, Farias P, Francisco R, Branco R, Simão FV, Gomes E, Pereira A, Vila MC, Fiúza A, Mortensen MS, Sørensen SJ, Morais PV. 2019. Tailings microbial community profile and prediction of its functionality in basins of tungsten mine. Sci Rep 9:19596. doi:10.1038/s41598-019-55706-6 31862994 PMC6925229

[5] Delgado-Baquerizo M, Oliverio AM, Brewer TE, Benavent-González A, Eldridge DJ, Bardgett RD, Maestre FT, Singh BK, Fierer N. 2018. A global atlas of the dominant bacteria found in soil. Science 359:320–325. doi:10.1126/science.aap9516 29348236

[6] Handelsman J. 2004. Metagenomics: application of genomics to uncultured microorganisms. Microbiol Mol Biol Rev 68:669–685. doi:10.1128/MMBR.68.4.669-685.2004 15590779 PMC539003

[7] Ferravante C, Memoli D, Palumbo D, Ciaramella P, Di Loria A, D’Agostino Y, Nassa G, Rizzo F, Tarallo R, Weisz A, Giurato G. 2021. HOME-BIO (sHOtgun MEtagenomic analysis of BIOlogical entities): a specific and comprehensive pipeline for metagenomic shotgun sequencing data analysis. BMC Bioinform 22:106. doi:10.1186/s12859-021-04004-y PMC8256542 34225648

[8] Riesenfeld CS, Schloss PD, Handelsman J. 2004. Metagenomics: genomic analysis of microbial communities. Annu Rev Genet 38:525–552. doi:10.1146/annurev.genet.38.072902.091216 15568985

[9] Soni R, Suyal DC, Sahu B, Phulara SC. 2021. Metagenomics: an approach to unravel the plant microbiome and its function, p 32–44. In Phytomicrobiome interactions and sustainable agriculture. John Wiley & Sons Ltd.

[10] Lu J, Rincon N, Wood DE, Breitwieser FP, Pockrandt C, Langmead B, Salzberg SL, Steinegger M. 2022. Metagenome analysis using the kraken software suite. Nat Protoc 17:2815–2839. doi:10.1038/s41596-022-00738-y 36171387 PMC9725748

[11] Nurk S, Meleshko D, Korobeynikov A, Pevzner PA. 2017. metaSPAdes: a new versatile metagenomic assembler. Genome Res 27:824–834. doi:10.1101/gr.213959.116 28298430 PMC5411777

[12] Wu Y-W, Simmons BA, Singer SW. 2016. MaxBin 2.0: an automated binning algorithm to recover genomes from multiple metagenomic datasets. Bioinformatics 32:605–607. doi:10.1093/bioinformatics/btv638 26515820

[13] Kang DD, Li F, Kirton E, Thomas A, Egan R, An H, Wang Z. 2019. MetaBAT 2: an adaptive binning algorithm for robust and efficient genome reconstruction from metagenome assemblies. PeerJ 7:e7359. doi:10.7717/peerj.7359 31388474 PMC6662567

[14] Alneberg J, Bjarnason BS, de Bruijn I, Schirmer M, Quick J, Ijaz UZ, Lahti L, Loman NJ, Andersson AF, Quince C. 2014. Binning metagenomic contigs by coverage and composition. Nat Methods 11:1144–1146. doi:10.1038/nmeth.3103 25218180

[15] Uritskiy GV, DiRuggiero J, Taylor J. 2018. MetaWRAP-a flexible pipeline for genome-resolved metagenomic data analysis. Microbiome 6:158. doi:10.1186/s40168-018-0541-1 30219103 PMC6138922

[16] Parks DH, Imelfort M, Skennerton CT, Hugenholtz P, Tyson GW. 2015. CheckM: assessing the quality of microbial genomes recovered from isolates, single cells, and metagenomes. Genome Res 25:1043–1055. doi:10.1101/gr.186072.114 25977477 PMC4484387

[17] Parks DH, Chuvochina M, Rinke C, Mussig AJ, Chaumeil P-A, Hugenholtz P. 2022. GTDB: an ongoing census of bacterial and archaeal diversity through a phylogenetically consistent, rank normalized and complete genome-based taxonomy. Nucleic Acids Res 50:D785–D794. doi:10.1093/nar/gkab776 34520557 PMC8728215

[18] Shaffer M, Borton MA, McGivern BB, Zayed AA, La Rosa SL, Solden LM, Liu P, Narrowe AB, Rodríguez-Ramos J, Bolduc B, Gazitúa MC, Daly RA, et al.. 2020. DRAM for distilling microbial metabolism to automate the curation of microbiome function. Nucleic Acids Res 48:8883–8900. doi:10.1093/nar/gkaa621 32766782 PMC7498326

[19] Peix A, Ramírez-Bahena MH, Velázquez E. 2018. The current status on the taxonomy of Pseudomonas revisited: an update. Infect Genet Evol 57:106–116. doi:10.1016/j.meegid.2017.10.026 29104095

